# The University of Michigan Dioxin Exposure Study: Predictors of Human Serum Dioxin Concentrations in Midland and Saginaw, Michigan

**DOI:** 10.1289/ehp.11779

**Published:** 2008-12-22

**Authors:** David H. Garabrant, Alfred Franzblau, James Lepkowski, Brenda W. Gillespie, Peter Adriaens, Avery Demond, Elizabeth Hedgeman, Kristine Knutson, Lynn Zwica, Kristen Olson, Timothy Towey, Qixuan Chen, Biling Hong, Chiung-Wen Chang, Shih-Yuan Lee, Barbara Ward, Kathy LaDronka, William Luksemburg, Martha Maier

**Affiliations:** 1 Department of Environmental Health Sciences and Risk Science Center, University of Michigan School of Public Health, Ann Arbor, Michigan, USA;; 2 Survey Research Center, Institute for Social Research, University of Michigan, Ann Arbor, Michigan, USA;; 3 Department of Biostatistics, University of Michigan School of Public Health, Ann Arbor, Michigan, USA;; 4 Department of Civil and Environmental Engineering, University of Michigan College of Engineering, Ann Arbor, Michigan, USA;; 5 Survey Research and Methodology Program, University of Nebraska–Lincoln, Gallup Research Center, Lincoln, Nebraska, USA;; 6 Vista Analytical Laboratory, El Dorado Hills, California, USA

**Keywords:** epidemiology, exposure pathways, polychlorinated biphenyls, polychlorinated dioxins, polychlorinated furans, soil contamination

## Abstract

**Background:**

We conducted a population-based human exposure study in response to concerns among the population of Midland and Saginaw counties, Michigan, that discharges by the Dow Chemical Company of dioxin-like compounds into the nearby river and air had led to an increase in residents’ body burdens of polychlorinated dibenzofurans (PCDDs), polychlorinated dibenzofurans (PCDFs), and dioxin-like polychlorinated biphenyls (PCBs), here collectively referred to as “dioxins.”

**Objectives:**

We sought to identify factors that explained variation in serum dioxin concentrations among the residents of Midland and Saginaw counties. Exposures to dioxins in soil, river sediments, household dust, historic emissions, and contaminated fish and game were of primary interest.

**Methods:**

We studied 946 people in four populations in the contaminated area and in a referent population, by interview and by collection of serum, household dust, and residential soil. Linear regression was used to identify factors associated with serum dioxins.

**Results:**

Demographic factors explained a large proportion of variation in serum dioxin concentrations. Historic exposures before 1980, including living in the Midland/Saginaw area, hunting and fishing in the contaminated areas, and working at Dow, contributed to serum dioxin levels. Exposures since 1980 in Midland and Saginaw counties contributed little to serum dioxins.

**Conclusions:**

This study provides valuable insights into the relationships between serum dioxins and environmental factors, age, sex, body mass index, smoking, and breast-feeding. These factors together explain a substantial proportion of the variation in serum dioxin concentrations in the general population. Historic exposures to environmental contamination appeared to be of greater importance than recent exposures for dioxins.

Polychlorinated dibenzo-*p*-dioxins (PCDDs), dibenzofurans (PCDFs), and coplanar polychlorinated biphenyls (PCBs), collectively referred to as dioxin-like compounds or diox-ins, are of concern because they are toxic, persist in the environment, have the potential for accumulation in the food chain, and are detected at low concentrations in virtually all humans [U.S. Environmental Protection Agency (EPA) 2003a, 2003b, 2003c]. A number of environmental exposure incidents have resulted in increased body burdens of dioxins, including the release of 2,3,7,8-tetra-chlorodibenzo-*p*-dioxin (TCDD) from a reactor accident in Seveso, Italy, in 1976 (Bertazzi et al. 2001; Landi et al. 1998); exposure to Agent Orange from the Vietnam conflict among the Ranch Hands cohort (Akhtar et al. 2004) and Vietnamese civilians (Baughman and Meselson 1973; Dwernychuk et al. 2002; Schecter et al. 1986, 2003); and victims of the Yusho (Masuda 2003; Rappe et al. 1978) and Yucheng (Guo and Yu 2003) rice oil poisoning incidents in 1968 and 1979, respectively. These studies have documented increased body burdens of dioxins among exposed populations but have provided limited data regarding the potential exposure pathways by which the dioxin releases have reached the human population. It is important to identify the pathways by which historic contamination continues to contribute to human body burdens and those that do not.

The pathways by which dioxins in environmental media reach humans are of particular interest in settings where there has been substantial environmental contamination over long periods of time. For the purposes of this article, we use the term “contamination” to mean the presence of dioxins above background levels in environmental media, where we define “background” as the concentration that would occur in an area that is without known point sources of that substance (U.S. EPA 2003d). The Dow Chemical Company facilities in Midland, Michigan, which have been in operation for > 100 years, have included chloralkali operations (electrolytic reduction of brine into chlorine gas and sodium hydroxide) in the early part of the 20th century, chemical waste incinerators in operation into the 1980s, and chlorophenol production between 1937 and 1980 (Burns et al. 2007). Soils in areas downwind of the Dow plant have elevated dioxin-like compounds with a congener pattern that is rich in PCDDs, and sediments in the Tittabawassee River downstream of the Dow plant have elevated levels with a congener pattern that is rich in PCDFs (Hilscherova et al. 2003). We conducted a human exposure study [University of Michigan Dioxin Exposure Study (UMDES)] in response to concerns among the population of Midland and Saginaw counties that the discharge by Dow of dioxin-like compounds into the river and air had led to an increase in residents’ body burdens of PCDDs, PCDFs, and PCBs. This question is central to the discussion of the circumstances and exposure pathways by which dioxin emissions may affect the human population.

## Methods

To investigate the factors that predict residents’ body burdens, 946 people were sampled from five geographically defined populations using a two-stage area probability household sample design. The issues of particular concern were whether living on contaminated soil, having contaminated household dust, and eating foods from the contaminated areas contributed to people’s body burden of dioxin-like compounds.

Adults ≥ 18 years of age who had lived in their current residence for ≥ 5 years were eligible to participate. Eligible subjects were randomly selected from the populations of five counties in Michigan and invited to complete an interview, donate an 80-mL whole blood sample, have their household dust collected, and have their soil sampled. All participants provided written informed consent, and all aspects of the study were approved by the University of Michigan institutional review board. We chose three counties (Midland and Saginaw, and part of Bay County) because of their proximity to the Dow Chemical Company and two counties (Jackson and Calhoun) approximately 200 km away as a reference population. Serum, household dust, and soil were analyzed for the 29 congeners recognized by the World Health Organization (WHO) as having dioxin-like activity (Van den Berg et al. 2006). We calculated the total dioxin-like toxic equivalent (TEQ) for each sample by multiplying the congener concentration by its toxic equivalency factor and summing these products. Samples that were less than the limit of detection (LOD) were estimated using 
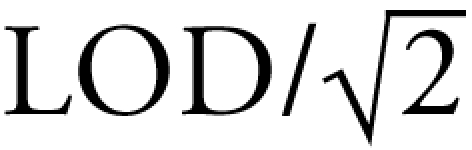
 (Finkelstein and Verma 2001). All serum results were lipid adjusted and survey weighted to reflect the entire referent population region. The interview is described in detail by Garabrant et al. (2009). Among other things, we asked subjects about consumption of specific fish species (bottom-feeding: catfish, carp, bullhead, and suckers; fish that live in the water column: walleye, perch, pike, pickerel, muskellunge, bass, trout, salmon, and steelhead; “pan fish”: bluegills, sunfish, and crappie; and other freshwater fish that are small enough to fit in a small pan and are legal to catch), where the fish were obtained (focusing on the contaminated areas of the Tittabawassee River, Saginaw River, and Saginaw Bay), and the number of meals consumed. The interview was designed to determine all species of fish that are commonly caught in the study regions. We used multiple imputation procedures [using IVEware software (University of Michigan, Ann Arbor, MI) with five imputations] to impute missing values in explanatory variables (Raghunathan et al. 2001).

We used linear regression models, using a stepwise variable selection procedure, to identify significant predictors of the log_10_ serum dioxin concentration. Five imputed data sets were used in each step, adjusted for all sample design features (including sampling weights, stratification, and clustering). In the first phase, age, age^2^, body mass index (BMI), sex, months of breast-feeding, pack-years of smoking, region of current residence, dioxin concentration in the top 1 in. (2.5 cm) soil around the house perimeter, and dioxin concentration in the garden soil were forced into the model. These factors were either important predictors from preliminary models or addressed the principal hypotheses under study. We then fit a model on the forced-in variables, allowing the variable (selected from the 114 other potential variables) with the smallest combined *p*-value to enter at each step, and refitted the-model. We calculated the combined *p*-value using Rubin’s multiple imputation combining rule, averaging the regression coefficients and accounting for between- and within-imputation variation (Rubin 1987). Variables once entered might be dropped if they were no longer significant as other variables were added. The stepwise variable selection procedure continued until the combined *p*-values-for all of the variables in the model (except for those forced in) were significant (*p* < 0.05) and none of the variables excluded from the model satisfied the significance level for entry into the model. Potential variables included soil dioxin concentrations, household dust dioxin concentrations, recreational activities in the contaminated areas, occupations (focused on those with potential dioxin exposure), diet (especially sport-caught fish, game meat, eggs, poultry, diary, and vegetables raised in contaminated soil), and property-use factors (e.g., backyard burning).

In the second phase, only age and sex were forced into the model (the other eight variables forced in the first phase were allowed to drop out), and stepwise selections were made from the variables selected in the first phase, plus age^2^ and an age × sex interaction term. After finding the variables list from this phase, we tested interaction terms (female × pack-years, BMI × age, BMI × sex, BMI loss × age, and BMI loss × sex). All analyses were performed using SAS, version 9.12 (SAS Institute Inc., Cary, NC). Separate regression models were created for the TEQ and for each of the five specific congeners that are the greatest contributors to the TEQ in serum (Table 1) using the data from all five regional populations combined. TCDD, 1,2,3,7,8-pentachloro-dibenzo-*p*-dioxin (1,2,3,7,8,9-PeCDD), and 1,2,3,6,7,8-hexachlorodibenzo-*p*-dioxin (1,2,3,6,7,8-HxCDD) made up 9%, 27%, and 20%, respectively, of the TEQ in the serum samples. 2,3,4,7,8-Pentachlorodibenzofuran (2,3,4,7,8-PeCDF) made up 9%, and PCB-126 made up 10% of the TEQ.

To assess the effect of an individual observation on each estimated parameter of the fit-ted model, we calculated for each observation the dfbeta diagnostic (the standardized difference in the parameter estimate due to deleting the observation). We retained in the models explanatory factors that were statistically significant (*p* < 0.05) when all observations were included, but became non significant when three or fewer observations were omitted; however, these are not presented in Table 2. We felt that these factors were unstable because their inclusion depended on, in many cases, a single influential observation. We examined explanatory factors for collinearity using variance inflation factors. A small number of factors that exhibited collinearity were recentered or transformed into categorical variables as needed to remedy this.

## Results

The models explained a large percentage of variation in serum dioxin concentration (Table 1): 70% for the TEQ, 63–67% for the PCDD and PCDF congeners, and 49% for PCB-126. The largest part of the variation (31–44%) was explained by what we have labeled demographic factors: age, age^2^, sex, BMI, BMI loss in the past 12 months, breast-feeding, number of pregnancies, race, smoking, and interaction terms. Soil and household dust dioxin content explained only a small part of the variation in serum dioxin levels: 0.5% for TCDD, 1% for PCB-126, and < 0.01% for the other congeners. Fishing and fish consumption explained 0.5–3% of the variation in serum dioxin concentrations, and meat, dairy, game consumption, and hunting explained < 1%.

Table 2 presents the parameter estimates for the factors that were statistically significantly associated with serum dioxin concentrations. For each congener, we adjusted the results in Table 2 for all other factors in the model. Because the regression models were calculated as log_10_(serum concentration), the parameter estimates are presented as 10^β^ for ease of interpretation. For continuous variables such as age, BMI, pack-years of smoking, and months of breast-feeding, we multiplied the serum dioxin concentration by 10^β^ for each unit increase in the variable. For categorical variables, such as race, sex, and current smoking, we multiplied the serum dioxin concentration by 10^β^ for a person who has that factor, compared with a person without the factor (i.e., a person at the reference level). When 10^β^ > 1.0, this indicates that the serum dioxin concentration increases as the factor increases, whereas when 10^β^ < 1.0, this indicates that the serum dioxin concentration decreases as the factor increases. Age was strongly associated with serum TEQ and with all serum congener concentrations. In addition, age^2^ was negatively associated with serum TEQ, 1,2,3,6,7,8-HxCDD, and 2,3,4,7,8-PeCDF, indicating that the relationship between age and serum concentrations was slightly less than exponential for these dioxins.

**Figure 1:**
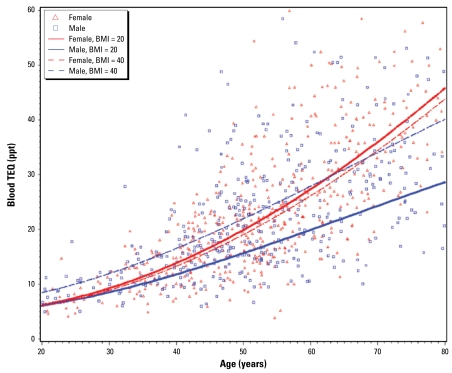
Serum TEQ by age among 946 UMDES participants, with predicted values by age, sex, and BMI. Blood TEQ values are shown only up to the 95th percentile to prevent compression of the scale by outliers.

The prediction model for TEQ showed a number of important relationships (Table 2). Females on average had higher TEQs than males, and the TEQ increased with age more dramatically among females than among males (Figure 1). Higher BMI was associated with higher TEQ in men but not in women. Breast-feeding was inversely associated with TEQ: As the length of breast-feeding increased, a woman’s TEQ decreased. The number of pack-years of smoking was also inversely associated with TEQ, indicating that as smoking increased, TEQ decreased.

The prediction model for TCDD showed that the TCDD concentration increased with both older age and female sex, and that females had greater increases in TCDD than males with increasing age (Table 2). Smoking was inversely associated with TCDD, indicating that as smoking increased, TCDD decreased. White race was associated with lower TCDD concentrations compared with other races. Although TCDD was the only dioxin for which we found an association with race, the study population was 93% white, resulting in low power to detect race effects.

The prediction model for 1,2,3,7,8-PeCDD showed that females on average had higher TEQs than males, and the TEQ increased with age more dramatically among females than among males. Breast-feeding was inversely associated with 1,2,3,7,8-PeCDD: as breast-feeding increased, a woman’s 1,2,3,7,8-PeCDD decreased. The prediction model for 1,2,3,6,7,8-HxCDD showed associations with age, age^2^ (negative), female sex, BMI, and a negative interaction between BMI and female sex (Table 2). Breast-feeding was inversely associated with 1,2,3,6,7,8-HxCDD, indicating that as breast-feeding increased, a woman’s 1,2,3,6,7,8-HxCDD level decreased. The number of incomplete pregnancies (self-reported stillborn, aborted, miscarried, or ectopic pregnancies) was positively associated with 1,2,3,6,7,8-HxCDD.

The prediction model for 2,3,4,7,8-PeCDF showed positive associations with age, female sex, BMI loss during the past year, and the interaction between age and sex. We found negative associations with age^2^ and breast-feeding. The prediction model for PCB-126 showed positive associations with age and BMI loss in the past year. We found negative associations with female sex, smoking, and the interaction between BMI and sex.

Residence in Midland and Saginaw counties was examined in three different historic periods, 1940–1959, 1960–1979, and 1980–2005, with the duration of residence during each period handled as a continuous variable. Residence in the area during 1960–1979 was positively associated with TEQ, TCDD, and 1,2,3,7,8-PeCDD, but not with other congeners. These results suggest that having resided in the area during a period 25–45 years before this study was associated with higher serum TEQ, TCDD, and 1,2,3,7,8-PCDD levels and are consistent with historic contamination from the Dow facility. Neither residence in 1940–1959 nor residence in 1980–2005 was associated with any congener. The emissions pattern in the 1940–1959 period is not known. After adjustment for all other factors in the model, including historical residence in Midland or Saginaw counties as described above, living currently in the floodplain or near the floodplain of the Tittabawassee River or in the plume area downwind of the Dow facility was not associated with either TEQ or any of the PCDD or PCDF congeners. We found a significant positive association for serum PCB-126 and living currently in the near floodplain compared with living in Jackson/Calhoun counties. However, we are not aware that PCB-126 was manufactured or used at Dow. A number of large iron foundries on the Saginaw River are known sources of PCB contamination in the sediments of that river.

A number of property-use factors were associated with increased serum dioxins. Having lived on a farm in 1940–1959 (whether it was in the Midland/Saginaw area or elsewhere), was associated with higher TEQ, 1,2,3,6,7,8-HxCDD, 2,3,4,7,8-PeCDF, and PCB-126. Although these results are consistent across most of the dioxins, the meaning is unclear. Trash burning on one’s property was associated with higher TCDD but not other congeners.

Using weed killers in 1940–1959 was inversely associated with 2,3,4,7,8-PeCDF but not other congeners, and not in other historic periods. We asked this question to pursue the hypothesis that use of phenoxyacetic acid herbicides [2,4-dichlorophenoxyacetic acid (2,4-D), 2,4,5-trichlorophenoxyacetic acid (2,4,5-T)] in the past would be associated with exposure to PCDDs. This hypothesis is not clearly supported by the data.

Even though 6% of the subjects reported ever having worked at Dow, we found little evidence that such work was associated with increased serum dioxin levels. Of those who reported working at Dow, 17% reported working with chlorophenol, 8% with Agent Orange, 8% with other Vietnam era herbicides, 15% with pentachlorophenol, 13% with 2,4-D, and 17% with 2,4,5-T. Work at Dow in 1940–1959 was positively associated with TCDD; however, working at Dow in 1980–2005 was inversely associated with 1,2,3,6,7,8-HxCDD and 2,3,4,7,8-PeCDF. None of the other variables that explored work at Dow, work with chlorophenol derivatives, or likely occupational exposure to dioxins was associated with increased serum dioxins.

A principal focus of our study was to assess activities that might involve contact with contaminated soils, river sediments, and household dust. These included living on contaminated soil, living with contaminated household dust, and pursuing activities in the contaminated water bodies and flood-plain (boating, swimming, picnicking, hiking, etc.). We found little evidence in the general population that these activities were associated with increased serum dioxins. Participating in water activities on the Tittabawassee River more than once per month in 1980–2005 was associated with increased TCDD, but less frequent activities and activities before this period were not. We found no relationship between these activities and any other dioxin in any historic era. Water activities in 1980–2005 on other rivers outside the contaminated area were inversely associated with 1,2,3,6,7,8-HxCDD but not other dioxins. The Saginaw River (which is formed in part from the Tittabawassee River) is also contaminated with PCDDs, PCDFs, and PCBs and is of concern (Michigan Department of Environmental Quality 2006). We found no evidence that water activities on the Saginaw River or in its floodplain were associated with increased serum dioxins.

We explored soil contamination in detail. Six different soil samples were assessed for each house and for each congener, as well as the TEQ: house perimeter top 1 in. (2.5 cm; the vegetation having been discarded), house perimeter 1–6 in. (2.5–15 cm), garden soil, near-river top 1 in., near-river 1–6 in., and the maximal contamination found in any soil sample on the property. Samples were stratified so that the top 0–1 in. (0–2.5 cm) sample could be examined separately from the 1–5 in. (2.5–15 cm) sample because surficial deposition of aerosols would be expected to affect only the top 1–2 cm of soil, whereas contamination from other pathways (e.g., fluvial deposition in the river floodplain) would be expected to affect deeper soil. We found soil contamination was significantly associated with serum dioxins in two instances.

The TCDD concentration in garden soil was statistically significantly associated with higher serum TCDD. This association was due to a single observation that was highly influential. When we included this observation in the regression analysis, we found a statistically significant association between garden soil TCDD and serum TCDD (parameter estimate = 1.012 ppt TCDD in serum/ppt TCDD in soil; *p* = 0.02). When we excluded this observation from the regression analysis, we found no statistically significant relationship (parameter estimate = 1.0016 ppt TCDD in serum/ppt TCDD in soil; *p* = 0.79). The observation appears to be valid and was not excluded from the regression model. The importance of garden soil TCDD concentration as a predictor of serum TCDD should be interpreted with caution insofar as the association depends on a single observation. Although there may be an association for high values of soil (based on a single influential data point at 140 ppt), for soil levels < 50 ppt TCDD (the range of the rest of the data), we found no significant association. The house perimeter top 1 in. (2.5 cm) soil PCB-126 concentration was associated with serum PCB-126 (parameter estimate = 1.001 ppt PCB-126 in serum/ppt PCB-126 in soil; *p* = 0.0002). This association was due to two influential observations. When we excluded these, we found no statistically significant relationship.

Even though no other soil variables entered in the stepwise models, we performed additional analyses of soil dioxins by forcing each of the soil variables into our final model. With the exception of results described above, no soil variable was statistically significantly associated with serum dioxins. We found no statistically significant associations between household dust dioxins and serum dioxins.

Consumption of fish after 1980, regardless of their source (store-bought, restaurant, or sport caught), was positively associated with TEQ, TCDD, and PCB-126. We found no positive associations between consumption of fish from the contaminated areas (Tittabawassee River, Saginaw River, or Saginaw Bay) and serum PCDD or PCDF levels. There were equivocal results for eating fish from the Saginaw River or Saginaw Bay in the past 5 years (positive association for consumption less than once per month, but a negative association for consumption of more than once per month). We found positive associations for serum 1,2,3,6,7,8-HxCDD and 2,3,4,7,8-PeCDF and consumption of walleye or perch from other sources (other water bodies, restaurants, and grocery stores). All other variables related to consumption of fish from the contaminated water bodies were either significantly negatively associated with serum dioxins or not associated. Overall, consumption of fish was positively associated with increased serum levels of a number of dioxins, but the findings were not related to fish from the contaminated areas, with the exception of PCB-126.

Consumption of store-bought meat more than once per week was positively associated with serum 2,3,4,7,8-PeCDF and 1,2,3,6,7,8-HxCDD. No other variable related to consumption of either game or commercial meat was associated with serum dioxins. We found no positive associations between serum dioxins and consumption of game from the contaminated areas.

Fishing in the Saginaw River and Saginaw Bay after 1980 was positively associated with serum TEQ, 1,2,3,7,8-PeCDD, and 2,3,4,7,8-PeCDF, and fishing in the Tittabawassee River between 1960 and 1979 was positively associated with 1,2,3,6,7,8-HxCDD.

We found no evidence that consumption of game meat from the contaminated areas was associated with increased serum dioxins. However, hunting in the areas around the Saginaw River or Saginaw Bay in 1960–1979 was associated with increased serum 1,2,3,7,8-PeCDD (2.3-fold) and 1,2,3,6,7,8-HxCDD (1.2-fold). Hunting in the area around the Tittabawassee River in 1960–1979 was associated with a substantial increase in serum TCDD (2.3-fold), but hunting in the same area after 1980 was associated with a substantial decrease (0.25-fold). Hunting in the contaminated areas after 1980 was not positively associated with any of the serum dioxins.

## Discussion

The overall pattern of findings in this population suggest little contribution from exposures in Midland and Saginaw counties during the last 25 years to serum dioxins from either contaminated soil, household dust, consumption of fish and game from contaminated areas, or recreational habits. The participants in our study had lived in their current residences for an average of 15–20 years, and those from Midland and Saginaw counties had lived in these counties for an average of > 40 years. Thus, it was a stable population with long-term exposure to the area and its contaminants. After taking into account age, sex, BMI, change in BMI, breast-feeding, and smoking, the largest contributor to both TCDD and 1,2,3,7,8-PeCDD was having lived in the Midland/Saginaw region in 1960–1979. A number of additional variables also suggested that historic exposures before 1980 contributed to serum dioxins: working at Dow in 1940–1959 (TCDD), fishing in the Tittabawassee River in 1960–1979 (1,2,3,6,7,8-HxCDD), hunting around the Tittabawassee River in 1960–1979 (1,2,3,7,8-PeCDD and 1,2,3,6,7,8-HxCDD), and hunting around the Saginaw River or Saginaw Bay in 1960–1979 (1,2,3,7,8-PeCDD and 1,2,3,6,7,8-HxCDD).

In contrast, we found little evidence that activities after 1980 contributed appreciably to serum dioxin levels. This is consistent with historic data on environmental dioxin measurements that show TEQ levels in many media increased beginning in the 1930s, peaked in the 1960s and 1970s, and have declined substantially over the past 35 years (Lorber 2002). We did find that water activities in the Tittabawassee River after 1980 were associated with increased TCDD; fishing on the Saginaw River or Saginaw Bay after 1980 was associated with increased TEQ, 1,2,3,7,8-PeCDD, and 2,3,4,7,8-PeCDF; and eating fish other than walleye and perch from the Saginaw River or Saginaw Bay in the past 5 years was associated with increased serum PCB-126. The adjusted *R*^2^ from all fish consumption and fishing activities combined was between 0.5% and 1% for the PCDD compounds, 2–3% for PCB-126, and 2–3% for 2,3,4,7,8-PeCDF, indicating that these explained a small amount of variation in the serum dioxins in this population. This should not be interpreted to mean that individuals who were heavy consumers of contaminated fish were unaffected; rather, it means that, on average, among the population the contribution was small.

Less than 1% of the general population of Midland and Saginaw counties ate bottom-feeding species (carp, catfish, bullhead, and suckers, which would be expected to have high levels of dioxins) from the contaminated areas, perhaps because there have been fish advisories against such consumption since at least the early 1980s. It is possible that heavy consumption of contaminated species could contribute to serum dioxin concentrations. In the Midland area, 2,3,4,7,8-PeCDF is of greatest interest with regard to this issue because it is present in high concentrations in Tittabawassee River sediments where bottom-feeding fish feed and it has a long half-life (7 years) (Milbrath et al. 2009) in humans. Our model suggests that fishing in the Saginaw River or Bay at least once per month since 1980 multiplies the serum 2,3,4,7,8-PeCDF concentration by a factor of 1.3 (an increase of 1.6 ppt compared with the median level of 5.4 ppt in Jackson and Calhoun counties). In contrast, consumption of fish from the contaminated areas was not associated with increased serum 2,3,4,7,8-PeCDF. It is possible that a larger sample of people who consumed contaminated fish regularly might have shown an association.

Walleye and perch are the most commonly consumed sport-caught fish from the Tittabawassee and Saginaw rivers and have low levels of dioxins compared with other species of local fish, likely because they are migratory species that spawn but do not reside in the contaminated areas. The association between eating fish from the Saginaw River or Bay and serum PCB-126 levels may be unrelated to Dow, because we are not aware that Dow produced or used this material in the Midland plant, and there are a number of foundries on the Saginaw River that have been historic sources of PCBs in the river.

Although our regression models did not find effects on serum dioxin levels from game or produce raised in the floodplain, we previously reported an individual who raised beef cattle in the floodplain and whose age- and sex-adjusted serum 2,3,4,7,8-PeCDF was the highest in our study (Franzblau 2008). This suggests that heavy consumption of meat raised on contaminated soil contributed substantially to serum 2,3,4,7,8-PeCDF.

Our findings showed no relationship between serum dioxins and either soil or household dust contamination. The single influential observation for garden soil TCDD was also the highest concentration (140 ppt) in our garden soil data. Soil TCDD contamination < 50 ppt showed no significant association with serum TCDD, nor did soil contamination contribute measurably to the serum levels of the other dioxin congeners we have evaluated. These findings are reasonably consistent with U.S. EPA (2003a) estimates of the relationship between tissue levels and TCDD intake from soil, which predict that a soil concentration of 13 ppt (the 95th percentile of the house perimeter 0–1 in. soil samples) would contribute 0.14 ppt to serum TCDD. Because we did not include children, this study should not be interpreted as providing reassurance about the risks of soil contamination to children. Our study included a substantial number of properties that were heavily contaminated: 118 properties had soil contamination of ≥ 90 ppt TEQ, and 21 properties had soil contamination of ≥ 1,000 ppt TEQ. The lack of evidence that contaminated household dust was associated with serum dioxin concentrations is also reassuring in that even though soil contamination contributes to household dust contamination, it does not contribute appreciably to the body burden of dioxins.

We compared the age- and sex-specific distribution of serum TEQ and PCDDs in our referent population (Jackson and Calhoun counties) with the data reported from the National Health and Nutrition Examination Survey (NHANES) 2001–2002 survey (Centers for Disease Control and Prevention 2001; Patterson et al. 2008). For the TEQ, the NHANES population 90th percentile value was 24% higher than in Jackson/Calhoun counties. For example, for a 50-year-old white male, the predicted 90th percentile TEQ was 25.4 ppt in the NHANES population versus 31.5 ppt in the Jackson/Calhoun population (Chen et al. 2008), indicating that our referent population distribution is reasonably similar to the U.S. general population.

Although other large data sets provide estimates of the distribution of serum dioxin levels, ours has the unusual advantage that it was gathered from a population-based sample using complex survey methods, and the inferences are applicable to the general population from which the sample was drawn. It also provides valuable insights into the complex relationships between serum dioxins and age, sex, BMI, BMI change, smoking, and breast-feeding. These factors together explain a substantial proportion of the variation in serum dioxin concentrations in the general population. This study also indicates that the contributions of direct environmental exposures, such as those we studied in Midland and Saginaw counties, cannot be properly assessed without careful control for the major explanatory variables such as age, sex, BMI, smoking, and breast-feeding. The contributions of environmental exposures to serum dioxin concentrations were small, compared with the demographic factors, in terms of their ability to explain variation.

This study was large and was capable of finding small associations that are statistically significant. Inferences regarding these associations should consider not only the statistical significance of the parameter estimate but also the magnitude of the effect and the amount of variance in serum TEQ or dioxins explained by the factor. Several of the significant findings described here both are small in magnitude and explain little variation in serum TEQ. Additional analyses of the upper tail of the distribution of serum dioxin concentrations are in progress using logistic regression methods, which we expect will provide insight into factors that predict having very high serum levels. Additional analyses of patterns of fish consumption incorporating analyses of the levels of contamination in various fish species from the contaminated waters would further refine our understanding of the contributions of fish contaminants to serum dioxin levels.

## Figures and Tables

**Table 1:** Adjusted *R*^2^ indicating the percentage of the variation in serum dioxin explained by the full regression model and by categories of variables.

Contribution to adjusted *R*^2^ (%)^a^	WHO 2005 TEQ^b^	TCDD	1,2,3,7,8-PeCDD	1,2,3,6,7,8-HxCDD	2,3,4,7,8-PeCDF	PCB-126
Overall (full model)	70.26	63.68	67.13	63.07	65.10	48.90
Demographic factors	39.63	30.48	44.24	36.52	40.13	31.23
Residence factors	0.55	3.40	1.09	0.00	0.00	0.37
Soil/household dust	0.00	0.51	0.00	0.00	0.00	0.96
Property-use factors	1.34	1.00	0.21	0.34	2.24	2.35
Work history factors	0.18	1.82	0.92	0.74	0.78	0.68
Water activities factors	0.42	0.57	0.00	0.61	0.70	0.77
Fish consumption and fishing	1.02	0.47	0.92	1.09	2.78	2.04
Meat and dairy consumption and hunting	0.00	0.34	0.82	0.18	0.17	0.16

The adjusted *R*^2^ would be decreased by the amount listed if the factor was removed from the full regression model.

a See Table 2 for a list of the factors included in each category.

b TEQs combined for the 29 congeners recognized by the WHO as having dioxin-like activity (Van den Berg et al. 2006).

**Table 2:** Linear regression results (stable predictors only) for TEQ and five congeners that are greatest contributors to TEQ.^a^

	Linear regression models											
	TEQ_DFP29-2005_^b^		TCDD		1,2,3,7,8-PeCDD		1,2,3,6,7,8-HxCDD		2,3,4,7,8-PeCDF		PCB-126	
Parameter	10^β^	*p*-Value	10^β^	*p*-Value	10^β^	*p*-Value	10^β^	*p*-Value	10^β^	*p*-Value	10^β^	*p*-Value
Demographic factors												

Age at interview – 50 years^c^	1.0249	0.00	1.0226	0.00	1.0213	0.00	1.0253	0.00	1.0222	0.00	1.0320	0.00
(Age at interview – 50 years)^2^^c^	0.9998	0.01					0.9997	0.00	0.9998	0.00		
BMI – 28 (kg/m^2^)^c^	1.0153	0.02			1.0121	0.01	1.0113	0.05				
BMI decrease in the past 12 months			1.0443	0.00					1.0379	0.00	1.0487	0.01
Total no. of months all children were breast-fed	0.9932	0.00	0.9892	0.00	0.9922	0.00	0.9923	0.00	0.9941	0.00		
Current smoker (yes vs. no)											0.7596	0.01
Sex (1 for female, 0 for male)	1.0692	0.11	1.3250	0.00	1.0912	0.04	1.0311	0.49	1.0289	0.51	0.9567	0.55
Hispanic or Latino (yes vs. no)											1.2823	0.02
Pack-years of smoking	0.9954	0.00	0.9930	0.01							0.9880	0.00
No. of pregnancies without giving birth							1.0562	0.00				
White race			0.7134	0.00								
(BMI – 28) × sex	0.9827	0.01			0.9843	0.01	0.9824	0.01				
Sex × (age at interview – 50 years)	1.0077	0.00	1.0191	0.00	1.0096	0.00			1.0096	0.00		

Residence factors												

No. of years lived in Midland/Saginaw in 1960–1979	1.0066	0.01	1.0269	0.00	1.0090	0.00						
Region: reside in near floodplain											1.2472	0.00

Property-use factors												

No. of years lived on a farm or property where crops, livestock, or poultry were raised in 1940–1959	1.0138	0.00					1.0093	0.02	1.0114	0.00	1.0314	0.00
No. of years lived on property where trash or yard waste was burned in 1940–1959			1.0222	0.00								
No. of years used weed killers on the property in 1940–1959									0.9831	0.01		

Work history												

No. of years worked at Dow in 1940–1959			1.0522	0.00								
No. of years worked at Dow after 1980							0.9867	0.01	0.9885	0.00		
No. of years served as emergency responder in 1940–1959			1.1597	0.00								
No. of years served as emergency responder in 1960–1979											0.9665	0.01
No. of years served as emergency responder after 1980			0.9517	0.00								

Water activities												

Did water activities in other river after 1980 (≥ 1 per month vs. never)							0.8584	0.01				
Did water activities in Tittabawassee River after 1980 (≥ 1 per month vs. never)			1.9577	0.00								

Fish consumption and fishing												

No. of years ate fish from any source after 1980	1.0072	0.00	1.0114	0.00							1.0178	0.00
Ate walleye or perch caught somewhere else, store-bought, or in restaurant during the last 5 years (≥ 1 per month vs. never)							1.1947	0.00	1.1933	0.00		
Ate walleye or perch caught somewhere else, store-bought, or in restaurant during the last 5 years (< 1 per month vs. never)							1.1476	0.01	1.2096	0.00		
Ate walleye or perch caught from the Saginaw River or Bay during the last 5 years (< 1 per month vs. never)					0.8319	0.00						
Ate any fish other than walleye or perch caught from the Saginaw River or Bay during the last 5 years (≥ 1 per month vs. never)	0.5571	0.00	0.6683	0.00			0.7093	0.00	0.5536	0.00	0.4077	0.00
Ate any fish other than walleye or perch caught from the Saginaw River or Bay during the last 5 years (< 1 per month vs. never)											1.2553	0.02
Did fishing activities in the Saginaw River or Bay after 1980 (≥ 1 per month vs. never)	1.2106	0.00			1.2785	0.00			1.3157	0.00		
Did fishing activities in the Saginaw River or Bay after 1980 (< 1 per month vs. never)					1.1708	0.00						
Did fishing activities in the Tittabawassee River in 1960–1979 (≥ 1 per month vs. never)							1.4277	0.00				

Meat consumption and hunting												

Did hunting activities in the surrounding areas of the Saginaw River or Bay in 1960–1979 (ever vs. never)					1.5381	0.00	1.2253	0.02				
Did hunting activities in the surrounding areas of the Saginaw River or Bay after 1980 (≥ 1 per month vs. never)					0.6994	0.00						
Did hunting activities in the surrounding areas of the Tittabawassee River in 1960–1979 (≥ 1 per month vs. never)			2.3135	0.00								
Did hunting activities in the surrounding areas of the Tittabawassee River after 1980 (≥ 1 per month vs. never)			0.2459	0.00							0.6170	0.00

a Unstable factors (where statistical significance depended on three or fewer observations) are not shown.

b TEQs of the combined 29 PCDD (D), PCDF (F), and PCB (P) congeners recognized by the WHO as having dioxin-like activity (Van den Berg et al. 2006).

c Age at interview was centered at 50 years, and BMI was centered at 28 kg/m^2^.
